# Transmembrane 29 *(Tmem29)*, a Newly Identified Molecule Showed Downregulation in Hypoxic-Ischemic Brain Damage

**DOI:** 10.3390/neurosci3010003

**Published:** 2022-01-01

**Authors:** Hing-Wai Tsang, Inderjeet Bhatia, Koon-Wing Chan, Godfrey Chi-Fung Chan, Patrick Ip, Pik-To Cheung

**Affiliations:** 1 Department of Paediatrics and Adolescent Medicine, Li Ka Shing Faculty of Medicine, The University of Hong Kong, Hong Kong, China; thwpaed@hku.hk (H.-W.T.); bi546@ha.org.hk (I.B.); kwchan@hku.hk (K.-W.C.); gcfchan@hku.hk (G.C.-F.C.); 2 Room 123, 1/F, New Clinical Building, Queen Mary Hospital, Hong Kong, China; 3 5/F, Virtus Medical Tower, 122 Queen’s Road Central, Hong Kong, China

**Keywords:** hypoxic-ischemic encephalopathy, *Tmem29*, downregulations, long non-coding RNA, nuclear protein, apoptosis

## Abstract

Transmembrane 29 (*Tmem29*) gene with unknown function is a gene located on the X chromosome of the mouse genome. The gene showed differential expression in the Vannucci neonatal hypoxic-ischemic mouse brain model. We found the gene expresses with different molecular forms, including a group of long non-coding RNA forming a family of transcripts. It was predominantly expressed in the testes, brain, and kidney of mouse. In vitro identification and functional characterization were carried out in Neuro2a cells. Using fluorescence microscopy, *Tmem29* protein was found to be constitutively expressed in mouse cell lines of different origins. Oxygen glucose deprivation (OGD) induced apoptotic cell death in Neuro2a cells and was confirmed by activations of caspase 3. *Tmem29* protein was found to be associated with cell death especially at the time points of caspase 3 activations. A similar response was obtained in glucose deprivation (GD) cultures suggesting *Tmem29* response to a common mechanism induced by OGD and GD. Downregulation of *Tmem29* was induced by OGD and GD, further validating its response to hypoxia-ischemia (HI) insults. Our findings contributed to further understanding of molecular events after hypoxic-ischemic insults and opens new avenues for developing protective and therapeutic strategies for hypoxic-ischemic encephalopathy or even pathological programmed cell death.

## 1. Introduction

Hypoxic-ischemic encephalopathy (HIE) is an acquired syndrome characterized by brain injury in neonates [1,2]. Detrimental outcomes of HIE depend on the severity and duration of the first insult and could be clinically classified into mild to severe HIE by various clinical parameters such as Sarnat staging or Thompson score [3,4]. HIE leads to a major neurological problem worldwide with around 1 million newborns dying from the disease [5]. HIE survivors often developed permanent neurological disorders including neuromotor impairment, intellectual disability, epilepsy, and cerebral palsy [6]. Many studies have shown that HIE causes complex biochemical and cellular damage including oxidative stress, nutrient deprivation, and calcium ion excitotoxicity [7,8,9]. However, our current knowledge fails to address effective therapeutic intervention that target HIE. As a result, there is a pressing need to explore more about the molecular mechanisms that occur after HI insults, which is critical for developing neuroprotective strategies for newborns who suffered from HIE.

Transmembrane 29 (*Tmem29*, Gene ID: 382245), previously annotated as chromosome X open reading frame 44-like (*CXorf44-like*) gene, is a protein-coding gene located on the X chromosome of the mouse genome. The gene expresses multiple splice variants, coding for different protein isoforms. The neighboring gene includes apurinic/apyrimidinic endonuclease 2 (Apex) 36.2 kbps upstream and methylphosphate capping enzyme pseudogene (Gm15105) 99.3 kbps downstream of *Tmem29*. According to the mouse genome database deposited in NCBI and Ensembl, no exon sharing was observed within these genes [10,11]. *Tmem29* homologs were deposited in the NCBI HomoloGene database and showed conserved protein sequence in chimpanzee (FAM156B, Gene ID:465646), dog (LOC607305, Gene ID: 607305), cow (LOC101907669, Gene ID: 101907669), and rat (LOC683684, Gene ID: 683684) [12]. The human ortholog of *Tmem29* annotated as Family with sequence similarity 104 member B gene (*FAM104B*, Gene ID: 90736) showed protein sequence conservations encoded by the 2nd exons in human and mouse transcripts. However, more complex regulations were observed in human ortholog with a higher number of splice variants and variations in the recruited exons from this locus.

Currently, minimal research has been investigated on *Tmem29*. A study conducted by Reinius et al. identified *Tmem29* as an X chromosome inactivation (XCI) escape gene by oligonucleotide microarrays and confirmed by RNA-FISH analysis, suggesting *Tmem29* was not a female-biased gene that commonly expressed on X chromosome [13]. Apart from this study, there is no current research in characterizing the gene or its association with a disease’s phenotypes, such as HIBD.

In this study, *Tmem29* was identified as HI-responsive gene by a differential gene expression analysis. We examined the mRNA and protein expression of this under-searched gene, and the cellular compartmentalization was predicted and experimentally presented in this study. The association of *Tmem29* with key cell death markers in HI-induced cell death was illustrated by an in vitro Oxygen Glucose Deprivation (OGD) and Glucose Deprivation (GD) model, offering crucial insights for further study on this gene. 

## 2. Materials and Methods

### 2.1. Differential Expressed Hypoxic-Ischemic Novel Molecules Analysis

Vannucci’s cerebral hypoxia-ischemia model was adopted to mimic the HI insult in neonates [14,15,16,17]. Six postnatal day 7 wildtype C57BL/6 mice in the HI group were subjected to unilateral carotid artery ligation and were exposed to 5% CO_2_/95% N_2_ in a hypoxic chamber for 2.5 h. The mice were sacrificed in post 20 hours’ time point as this corresponds to an active phase of evolving tissue damage with mixed degrees of cell death or pending cell deaths [14,15,16,17]. Clontech^®^ PCR-Select cDNA Subtraction Kits (Takara Bio Inc., Nojihigashi, Japan) was used to identify differentially expressed cDNA. In brief, total RNA was extracted from each of the two cerebral hemispheres of each mouse. Transcriptome comparison was achieved by cDNA libraries synthesized by poly(A)-RT PCR, restriction digested with RsaI (New England Biolabs, Ipswich, UK), and ligated with different 5′ ends adaptors, generating a tester cDNA (control) and driver cDNA (target sample) library. Two populations of cDNA were allowed to hybridize, and complementary sequences were removed. The remaining unhybridized cDNA represented gene transcripts that were differentially expressed after HI insults, which were amplified by PCR, sub-cloned, and identified by Sanger Sequencing. The differential expressed sequences were aligned to mouse genome by Nucleotide Basic Local Alignment Search Tool (BLAST) in NCBI, searching for homology sequence within genes or outside annotated gene regions. HI-responsive gene was concluded only from the consistently altered expression in all 6 tested mice. 

### 2.2. Mice Organ mRNA Expression Studies of Tmem29

Adult wild-type male C57BL/6 mice were sacrificed, and the organs were harvested and snap-frozen in liquid nitrogen. RNA was extracted from the tissues by homogenizing in 1 ml TRIzol^®^ Reagents (ThermoFisher Scientific, Waltham, MA, USA) per 50 mg tissue according to the manufacturer’s protocol. Extracted RNA was measured by NanoDrop^TM^ spectrophotometer (ThermoFisher Scientific) and the integrity was analyzed by A260/A280 ratio. One microgram of total RNA was used to reverse transcribe into cDNA by Superscript II Reverse Transcriptase (ThermoFisher Scientific). The expression level of *Tmem29* in each organ was measured by absolute quantification real-time PCR by TaqMan-based method. TaqMan probe was designed to target the conserved region of the novel gene with primers flanking the conserved and specific regions of different splice variants. The probe sequence was 5′FAM-ACGGTAGTTGTCATCCTC-MGBNFQ-3′ (ThermoFisher Scientific) and the sequences are listed in Table S1 (Supplementary Materials). The position of the primers relative to *Tmem29* transcripts are illustrated in Figure S1 (Supplementary Materials). The arithmetic estimation was performed using the following equations:

Total = S1 * + S2 ^#^+(nt-A1 + nt-C1 + nt-D)

* Sub-total 1 (S1) = (α-IIIa + β + γ + κ) + (nt-A2 + nt-B + nt-C2)

^#^ Sub-total 2 (S2) = (α-IIIb) + (nt-A3 + nt-C3)

The relative abundance of protein-coding and non-translatable transcripts was estimated by adding the measured abundance of all translatable transcripts and non-protein-coding transcripts to the total number of transcripts, measured by conserved exon 2 in the tested sample. Standard Curve (copy number per nano-gram of RNA) was obtained by serial dilutions of different molecular forms inserted TOPO pCR2.1 plasmid (ThermoFisher Scientific) and was used for quantification.

### 2.3. In Vitro Protein Expression Analysis of Tmem29

Neuro2a neuroblastoma cell line Neuro2a RRID: CVCL_0470, (American Type Culture Collection, ATCC, Manassas, VA, USA), NG108-15 RRID: CVCL_0464 (American Type Culture Collection, ATCC), EOMA RRID: CVCL_3507 (American Type Culture Collection, ATCC), and C2C12 RRID: CVCL_UR38 (American Type Culture Collection, ATCC) was grown in Dulbecco’s Modified Eagle Medium (DMEM) with 10% fetal bovine serum (ThermoFisher Scientific) and incubated at 37 °C in 5% CO_2_ atmosphere. Western blotting and immunostaining with custom-designed antibodies in a dilution of 1:1000 and 1:500 respectively was done. The custom-designed antibody was raised by Applied Biological Materials Inc. (Richmond, BC, Canada) and annotated as AP675, the batch no. of the immunized rabbits. The antibody was raised against the conserved NLS region of the protein ^27^PRSKRIKKDQDIQ^39^ as the antigen. The sequence was checked without full alignments with other known protein sequences by BLAST, to minimize the binding to non-specific antigens. Protein knockdown experiment was performed by delivery of short interference RNA (siRNA), named as siRNA1 (5′-gguguguguuguucauuccaccugu-3′), siRNA2(5′-gagagaguaccagaaagcagcuuaa-3′), and siRNA3(5′-acaacaucuugaaggaggcucauuu-3′) in cultures by using lipofectamine 2000 (ThermoFisher Scientific), and the target regions of *Tmem29* transcripts are illustrated in Figure S2 (Supplementary Materials). The transfection mixtures were allowed to incubate with OPTI-MEM (ThermoFisher Scientific) for 24 h in Neuro2a cells, and total RNA and protein lysates were collected for expression analysis.

### 2.4. Oxygen Glucose Deprivation in Neuro2a Cultures

Oxygen Glucose Deprivation (OGD) model was established by replacing the growth medium with N_2_ flushed/CO_2_ balanced glucose-free medium and incubating in hypoxic conditions. The culture was put into an air-tight chamber, connected to a 5% CO_2_/95% N_2_ gas supply. The chamber was placed into a 37 °C incubator for 24 h followed by cell death assessments. Quantification of cell viability and apoptotic cells by flow cytometry was done using Annexin V Apoptosis Detection Kit (BD Bioscience, Franklin Lakes, NJ, USA) (Figure S3, Supplementary Materials). Western blot analysis was done using 15 μg protein lysate per lane applied to 10% SDS polyacrylamide gel electrophoresis and transferred to nitrocellulose membrane. The blots were probed with an anti-mouse cleaved caspase 3 (Cell Signaling Technology, Danvers, MA, USA) and anti-mouse LC3A/B (Cell Signaling Technology) antibodies at different post-insult time points. Anti-mouse α tubulin (MilliporeSigma, St. Louis, MO, USA) was used as an equal loading control.

### 2.5. Statistical Analysis

All data were expressed as mean ± standard deviation with 3 independent replicate experiments. One-way ANOVA followed by a post Dunnett’s *t*-test was applied to the data testing for the mean expression differences between mock-transfected group and siRNA-treated cultures. An unpaired *t*-test was applied to test the cell death status in GD and OGD groups at the same time point. *p*-values lower than 0.05 were considered to be statistically significant. Analysis was performed by SPSS Statistics for Windows, Version 27.0 (IBM Corp., Armonk, NY, USA) and Prism for Windows, Version 8.01 (GraphPad Software, San Diego, CA, USA).

## 3. Results

### 3.1. Identification of a Differentially Expressed Gene after HIBD and Expressed as a Transcript Family

**Figure 1:**
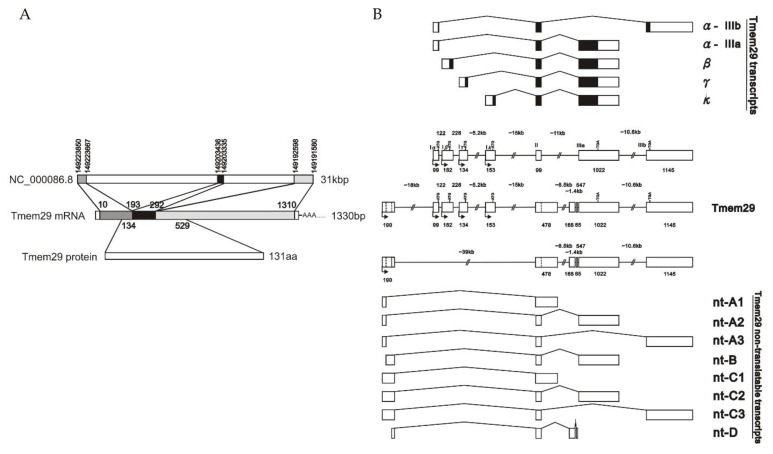
Genomic Structure of *Tmem29*. (**A**) *Tmem29* mRNA was composed of 3 exons, encoded by 3 clusters in mouse X chromosomes. *Tmem29* transcripts are predicted to encode a gene product with 131 amino acids. Numbers indicated the relative genomic or mRNA position of *Tmem29*; (**B**) *Tmem29* transcripts family showed a conserved 2nd exon of the transcripts. The non-coding transcripts recruited a more 5′ distal 1st exon than the protein-coding transcripts. Variations of exon numbers were commonly observed in non-translatable transcripts. Numbers indicated the exon length.

The genomic structure of the differentially expressed gene was summarized in Figure 1. Subtraction results revealed a primary sequence homology to a gene annotated with Transmembrane Protein 29 (*Tmem29*) in the NCBI database which was 1330 base pair in length, scattered with 3 clusters, over 31 k base pair in mouse X chromosome (NC_00086 REGION: complement, 149191580..149223854) (Figure 1A). In silico protein translation prediction showed an open reading frame (ORF) composed of 3 exons, encoding a product with 131 amino acids in length. 

Further genomic analysis of *Tmem29* revealed expression of multiple transcripts with both protein-encoding transcripts and lncRNA (Figure 1B). A total of 13 splice variants were identified through alignment with mouse Expression Sequence Tag (EST) and genomic database with the primary identified differentially expressed sequence (later annotated as *Tmem29*γ isoform). *Tmem29* was updated spanning 61 k base pair on X chromosome, forming a transcript family. Most of the protein-coding transcripts and lncRNA were composed of 3 exons, similar to the primary identified transcripts from the subtraction results. A conserved sequence, mostly the 2nd exon of the transcripts, was identified in both types of the splice variants. *Tmem29* lncRNA recruits the 1st exon more 5′ distal to those of the protein-coding version, and the exon numbers vary from 2–4 exons instead of 3 exons in the protein-coding transcripts. The transcripts were re-annotated and sub-grouped by the protein-coding transcripts or lncRNA. According to the relative genomic position of the recruited 1st exon and the transcripts’ structure, protein-coding splice variants named as α-IIIa (NM_001359038), α-IIIb (NM_001164684), β (NM_001164686), γ (XM_006528894), and κ (XM_030251377); lncRNA names as nt-A1 (EST:BY748377), nt-A2 (NR_152724), nt-A3 (MW266129), nt-B (NR_152725), nt-C1 (EST:CB601848), nt-C2 (EST:BM653242), nt-C3(EST:BY288280), and nt-D (EST:AA250694).

### 3.2. Complex Regulation of Tmem29: Expressed Differently in Various Adult Mice Organs

**Figure 2:**
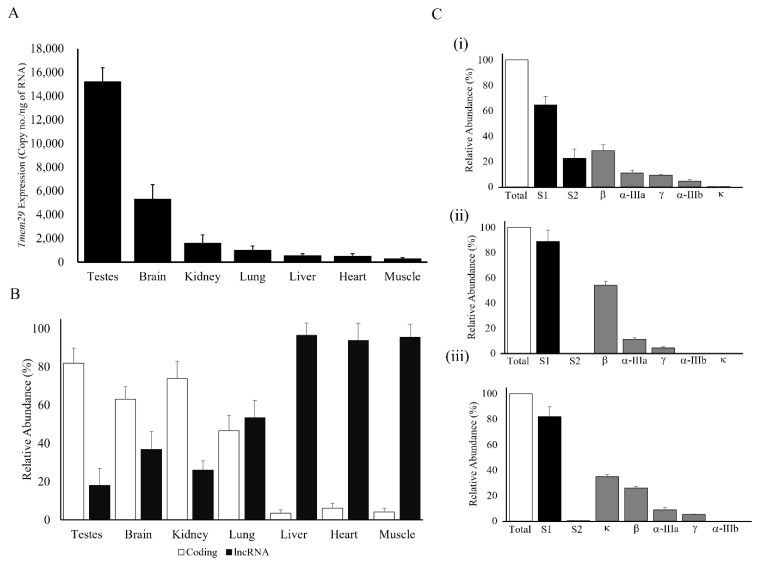
Expression profiling of *Tmem29* in different mice organs by Taqman absolute quantification PCR. (**A**) Quantification of Tmem29 transcripts in different adult wild-type mice organs; (**B**) Relative coding and lncRNA expression in different mice organs. Individual subgroup (S1, S2) and different splice variants’ relative abundance was referenced to total *Tmem29* mRNA and expressed in percentage. Relative abundance measurement of protein-coding transcripts in mice (i) brain, (ii) kidney, and (iii) testes; (**C**) Relative abundance measurement of protein-coding transcripts in mice (i) brain, (ii) kidney, and (iii) testes. Data were expressed in mean ± standard deviation with 6 individual mice, and experiments were done in triplicate.

Expression of *Tmem29* transcripts was studied by Taqman absolute quantification PCR in adult mice brains as well as in the other selected organs (Figure 2). Testes demonstrated the highest expression followed by brain and kidney. Mice lung, liver, heart, and muscle showed a relatively low expression level of *Tmem29* (Figure 2A). The proportion of the protein-coding transcripts and lncRNA were analyzed using primer sets based on the transcript structure which includes the common exon 2 and alternate 3rd exon of the transcripts (Figure 2A). *Tmem29* transcript expression varied among the brain and the selected organs (Figure 2B). In the brain, kidney, and testes, most *Tmem29* transcripts were coding transcripts. It is interesting to note that low *Tmem29*-expressing organs produce most non-translatable transcripts, which is a complementary phenomenon to that of the higher-expressing organs (Figure 2B). The expression pattern of the protein-coding transcripts was investigated in the high *Tmem29*-expressing organs to predict the predominant splice variants of the gene which may serve an important cellular function. The transcript β showed a relatively higher abundance when compared to other splice variants in the same organ. The relative abundance of transcripts β in the brain measured as 33.4 ± 7.3% (Figure 2C(i)), kidney 56.1 ± 8.7% (Figure 2C(ii)), and testes 27.1 ± 8.5% (Figure 2C(iii)). Moreover, a testes-specific and high-expressing transcript κ was also detected with a relative abundance of 37.7 ± 7.9%, with almost no expression found in other organs by the same detection method (Figure 2C(iii)).

### 3.3. Tmem29 Encodes for a Nuclear Protein

**Figure 3:**
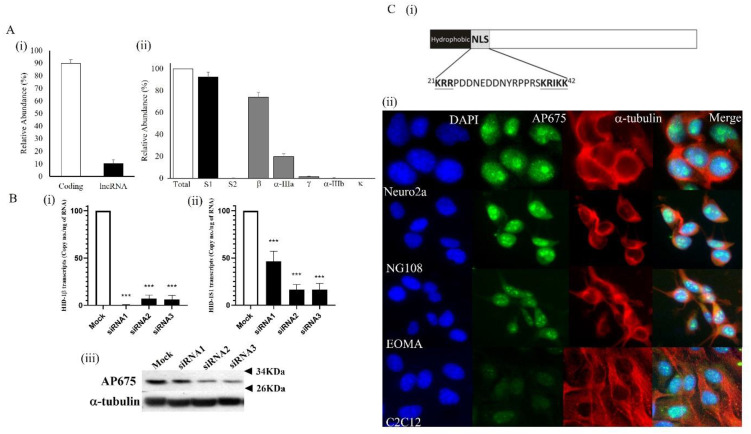
Characterization of *Tmem29* protein in mouse cell lines. (**A**) Characterization of *Tmem29* expression in Neuro2a cells by absolute quantification of (i) coding and lncRNA *Tmem29* transcripts, and (ii) different splice variants. Individual subgroup (S1, S2) and different splice variants’ relative abundance was referenced to total *Tmem29* mRNA and expressed in percentage; (**B**) Detection of *Tmem29* protein in siRNA knockdown Neuro2a cells. (i) Quantification of *Tmem29*β (ii) *Tmem29-*S1 subtype transcripts in Neuro2a cells. (iii) Immunoblot of AP675 in siRNA-transfected Neuro2a cell lysate. Data were expressed in mean ± standard deviation with 6 individual mice and experiments were done in triplicate. *** *p* < 0.001; (**C**) Cellular localization of *Tmem29* protein in Neuro2a, NG108, EOMA, and C2C12 cells. (i) Predicted NLS sequence of *Tmem29* protein. (ii) Representative immunostaining by AP675 in different cell lines. Morphology was captured at 400x magnification.

The gene expression and cellular localization were characterized by a custom-raised antibody (AP675) in a mouse neuroblastoma Neuro2a cell line. Quantification of *Tmem29* transcripts in Neuro2a cultures revealed expression of *Tmem29*, with predominantly expressing protein-coding transcripts, showing adequate expression of *Tmem29* in Neuro2a cells for characterization purposes (Figure 3A(i)). Aligning with previous results, *Tmem29*β was also found to be the predominant *Tmem29* isoform (Figure 3A(ii)). Characterization of the custom-raised antibody was evaluated by transfecting cells with short-interference RNA (siRNA), targeting to 5′-UTR of *Tmem29*β isoform as well as the S1 conserved region of *Tmem29* transcripts. The level of mRNA was found to be significantly suppressed (*p* < 0.001) by the 3 siRNAs by *Tmem29*β (Figure 3B(i)), and S1 subtype (Figure 3B(ii)) quantification methods with higher efficiency knockdown was observed by siRNA2 and siRNA3 transfection. The expression of *Tmem29* protein was successfully shown by AP675 antibody with a single specific band of size between 26 KDa and 34 KDa according to the pre-stained protein ladder in the non-transfected culture (Figure 3B(iii)). Higher efficiency siRNA2 and siRNA3 knockdown showed corresponding lower protein expression by AP675 immunoblotting in the transfected cultures (Figure 3B(iii)). In silico domains or motif identification of *Tmem29* protein revealed a potential nuclear localization signal (NLS) peptide sequence located near the N-terminal of the protein (Figure 3C(i)). The NLS sequence predicted by NLStradamus (revision r.9) was ^21^KRRPDDNEDDNYTPPRSKRIKK^42^ [18]. However, apart from the NLS, there were no other functional domains common to a known protein identified by the bioinformatics approach. To eliminate the possibility of cell-specific cellular localization, AP675 immunostaining was also performed in NG108, EOMA, and C2C12, in parallel with Neuro2a cells. Apart from Neuro2a cells, NG108, EOMA, and C2C12 also express *Tmem29* protein. Heterogenous positive AP675-FITC staining was obtained coherently with DAPI staining in all 4 testing cell lines (Figure 3C(ii)). Moreover, no positive staining could be obtained in the cell cytoplasm that was counter-stained with α-tubulin-TRITC in all 4 testing cell lines, indicating *Tmem29* protein was compartmentalized in the cell nucleus.

### 3.4. Downregulation of Tmem29 Protein after Oxygen Glucose Deprivation (OGD) and Glucose Deprivation (GD) in Neuro2a Cells

**Figure 4:**
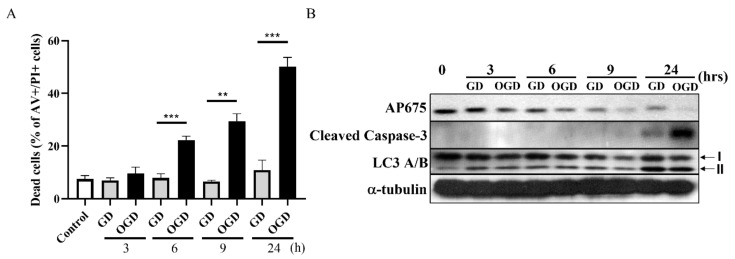
Downregulation of *Tmem29* protein upon OGD in Neuro2a cells. (**A**) Significant cell loss was obtained after 6, 9, and 24 h of OGD insults; (**B**) Representative protein expression analysis by Western blot revealed a progressive decrease in *Tmem29* protein expression by GD and OGD insult. The protein expression was lost at 24 h time point with a high abundance of cleaved caspase-3. Cultures responded to low glucose content with active autophagic marker LC3A/B expressed in both GD and OGD with highest expression at 24 h time points. Values were expressed as mean ± SD from 3 independent experiments with each sample done in triplicate. ** *p* < 0.01; *** *p* < 0.001.

In vitro *Tmem29* protein response to HI insults was demonstrated in Neuro2a cell cultures by OGD versus GD as a control (Figure 4). Significant time-dependent cell loss was observed with increasing incubation time under both OGD and GD conditions in Neuro2a cells (Figure 4A). Cleavage of caspase 3 as demonstrated by Western blotting was only seen at 24 hours’ time point, with a stronger intensity in OGD than in GD-treated cells (Figure 4B). LC3A/B-I form showed progressive downregulation during the time course of experimental OGD. However, a complementary upregulation was seen in active LC3A/B-II form with highest expression detected at 24 hours’ time point in cultures treated with both OGD and GD. *Tmem29* protein, detected by AP675, responded to both OGD and GD insult in a time-dependent manner and showed minimal expression at 24 h in OGD conditions.

## 4. Discussion

To our knowledge, we are the first group to characterize *Tmem29*, an understudied molecule that is differentially expressed in Vannucci neonatal mice hypoxic-ischemic model. *Tmem29* is localized in cell nuclei and was responsive to OGD insults in Neuro2a cells. To the current findings so far, the annotation of Transmembrane 29 (*Tmem29*) sequence data deposited in the NCBI database does not document any functions regarding a receptor of its predicted proteins, and there is still room to debate the name “transmembrane” annotated to this gene. 

Subtractive hybridization results showed *Tmem29* responded to HIBD and expressed different molecular forms including protein-coding and lncRNA. Criteria that were set to identify and validate the novelty of the clones after subtraction were: (1) The clones should be fully aligned in the mouse genome; (2) The clones should encode a sense gene product; (3) No functional domains matching, and documented functions in the predicted gene product; (4) No reports associated the encoded gene product to HIBD. Upon fulfillment of these criteria, *Tmem29* was decided for further investigations. Previous transcriptome analysis demonstrated a significant number of EST or known genes differentially expressed after HI insult and functionally participated in different cellular events [19,20,21,22,23]. Majority of the obtained genes were found to be upregulated, belonging to transcription factors and genes involved in metabolism whereas a minority of them showed downregulation such as heat shock cognate 70 (*Hsc70*) which was found constitutively expressed in unstressed cells and found neuroprotective with increased expression [24,25,26]. Moreover, genes related to stress and apoptosis were also captured. However, most of the identified genes in this category, such as peroxiredoxin (*Prdx6*) and heat shock protein 47 (*Hsp47*) were found to be upregulated after HI insults [24]. Thus, the identification of *Tmem29* and its association with HIBD in this study supplements a new gene in this category, specially showing downregulation after the insults. 

Expression studies of various organs were found to be informative. Although the gene was initially screened as differentially expressed in the brain, testes, and kidney, these organs also showed a relatively higher expression among the organs tested in this study. Organ-specific expression of *Tmem29* transcripts was also observed, such as testis-specific *Tmem29*κ, suggesting corresponding organ-specific regulation of this gene. 

*Tmem29* expresses lncRNA, and the transcripts have all been found to be complete transcripts with no additional exon-sharing with other genes. The presence of the non-translatable transcripts is coherent with previous findings suggesting that only 1/5 of human transcripts across the genome are translatable; the remaining 4/5 are non-translatable products such as lncRNA sequences [27]. A dominant expression of the non-coding transcripts was observed in low-expression organs such as the liver, heart, and muscle, suggesting an organ-specific regulation of the non-coding transcripts. According to the relative position of the lncRNA and protein-coding elements within the gene region, *Tmem29* non-coding transcripts were classified as sense-overlapping lncRNA as no anti-sense transcripts could be detected and sequenced from the locus in our experimental design so far [28]. Recent research identified HI-responsive long-coding RNA and its expression profile functions in regulating genes involved in basic signaling pathways such as NF-kappa B, toll-like receptor, and even providing neuroprotection [29,30,31]. Thus, the functions of *Tmem29* lncRNA cannot be ruled out as they may participate in important biochemical processes induced by HIBD.

Characterization of *Tmem29* protein was carried out by providing a basic understanding of the identified molecules in a cellular model. A self-raised anti-*Tmem29* antibody was generated for protein detection purposes with specificity and antigenicity checked. Single-band was shown by Western blot with decreased intensity in siRNA-transfected culture, suggesting the antibody was able to detect endogenous *Tmem29* protein. As detected by the self-raised antibody, *Tmem29* expresses in protein and was first identified as a nuclear protein. In silico protein motifs or domains’ prediction revealed a nuclear localization signal (NLS) domain in the predicted protein sequence and proved to be functional in different cell lines. Moreover, our results demonstrated *Tmem29* protein was constitutively expressed in unstressed cells. All the translatable transcripts of the gene carry the NLS domain, implying that all the gene products are potentially localized into the cell nucleus. The results from the cellular model also showed no positive staining in the cell cytoplasm, indicating the strict cellular compartmentalization enforced by the NLS on the protein, further postulating the functional importance of the gene inside the cell nucleus. 

The initial screening and identification of *Tmem29* was further validated by the in vitro OGD model. *Tmem29* was found progressively downregulated with increasing OGD incubation time in Neuro2a cultures. The protein downregulation coincided with the activation of cleaved caspase 3, suggesting *Tmem29* protein response to cell death, especially in the apoptotic pathway. Apart from that, *Tmem29* also responded to GD. Relatively mild decrease in *Tmem29* protein expression was observed during the time course of GD, which was associated with the caspase 3 activation, suggesting *Tmem29* response to a common inducer in OGD and GD. Previous studies demonstrated that neurons induced autophagic adaptive responses to counteract the stress induced by OGD and GD, which showed protective effects in regulating the cellular activity to restore physiological conditions [32,33,34]. The obtained increased level of LC3A/B II form throughout the time course in our study suggested that macro-autophagy was induced in OGD- and GD-treated Neuro2a cultures. *Tmem29* may play a role in sustaining cell functionality by contributing to the adaptive response to the insult, and the bio-effect should be diminished to promote cell death or apoptosis. Thus, the results in this study may offer a novel target molecule to improve the survival of the brain cells.

The exact identity and functions of *Tmem29* still need further elucidation and some knowledge gaps remained unexplored: (1) Apart from the NLS, there are no known protein motifs/domains identified in the protein sequence, which may provide further traceable direction in predicting the functional identity of the gene; (2) No evidence to rule out the presence of a novel motif and domain encoded by *Tmem29* with functional interactions with DNA, other proteins partners, and even homo- or hetero-complex formation; (3) The possibilities of multi-directional functions of this gene as *Tmem29* expresses both protein product and lncRNA. Thus, our preliminary results pave the way for further investigations to define a solid functional identity of the molecule.

## 5. Conclusions

In conclusion, our study newly identified *Tmem29* as a HI-responsive gene. The gene is expressed in multiple splice variants with the expression of organ-specific transcripts. *Tmem29* protein was proven to be constitutively expressed and identified as a nuclear protein. The protein expression was found to be downregulated in vitro OGD and GD insults, specifically associated with apoptotic cell death. These findings opened new possibilities in supplementing the current knowledge in HIBD and pathological programmed cell death.

## Data Availability

The data presented in this study are available on request from the corresponding author.

## Supplementary Materials

The following supporting information can be downloaded at: https://www.mdpi.com/article/10.3390/neurosci3010003/s1, Table S1: Primers for absolute quantification real-time PCR; Figure S1: Schematic diagram of primers used in absolute quantification real-time PCR; Figure S2: Schematics diagram of the relative position of siRNA on *Tmem29*β transcripts; Figure S3: Gating Strategy for apoptotic cells determination in OGD and GD treated Neuro2a cells.
