# Supplementary material for: Transmembrane 29 (Tmem29), a Newly Identified Molecule Showed Downregulation in Hypoxic-Ischemic Brain Damage
Source: NeuroSci. 2022 Jan 1;3(1):41–51. doi: 10.3390/neurosci3010003 (PMC11523738; doi:10.3390/neurosci3010003)
Supplement: Supplementary file 1 [file neurosci-03-00003-s001.zip › neurosci-1502742-supplementary.pdf]

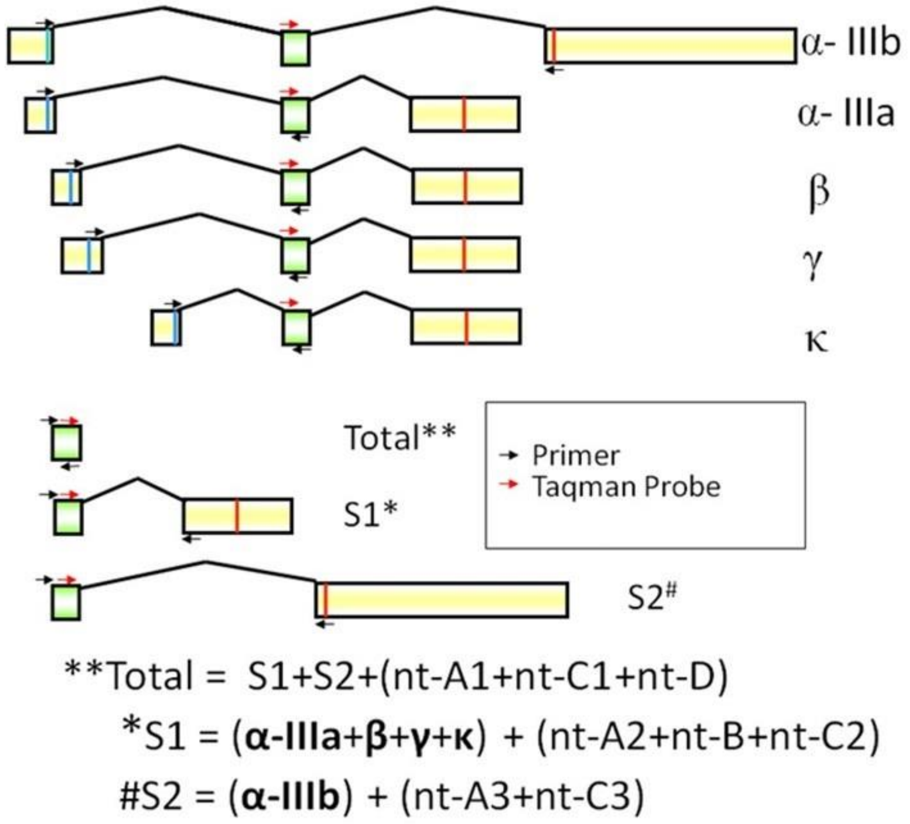

Figure S1. Schematic diagram of primers used in absolute quantification real-time PCR. Black arrows indicated the relative positions of the primers and red arrows indicated the position of the Taqman® probe which was targeted to different HID-1 transcripts. Blue lines showed the relative position of the start codon and red lines showed the stop codon which is predicted from the bioinformatics approach as a sense protein can be translated with this region of the transcripts.

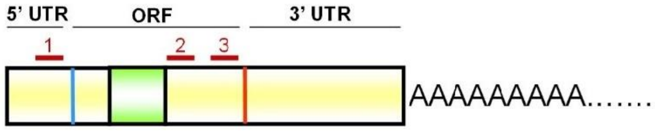

1	mHID1β -5UTR_30	β only
2	mHID1-ORFE3_294	Sub-total 1
3	mHID1-ORFE3_415	Sub-total 1

Figure S2. Schematics diagram of the relative position of siRNA on HID-1β transcripts. Red bars indicated the position of siRNA.

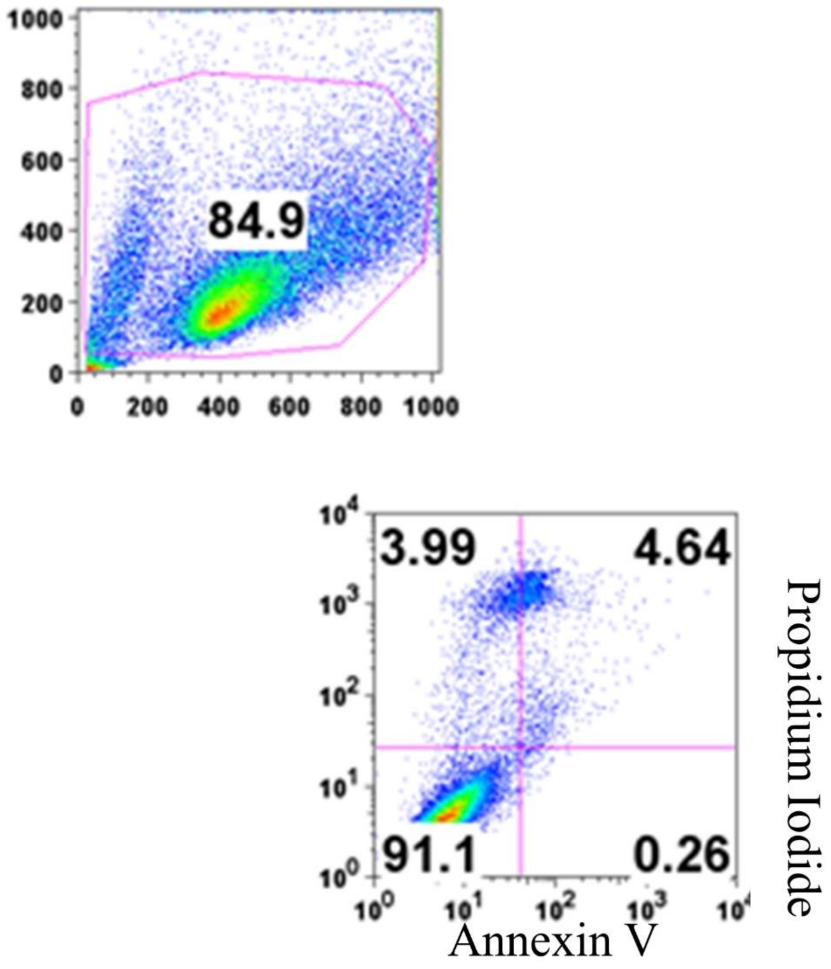

Figure S3. Gating Strategy for apoptotic cells determination by OGD in Neuro2a cells.

Table S1. Primers for absolute quantification real-time PCR.

Primer	Sequence (5' to 3')	Size (bp)
HID-1α-IIIa	Sense - tgaaccagccaatccttaacctaa	171
	Antisense - ttcctttcattatcactgcttg	
HID-1α-IIIb	Sense - tgaaccagccaatccttaacctaa	193
	Antisense - ctaggcgagagctaggaggga	
HID-1β	Sense - gtgcggtgtgtgttgttcat	178
	Antisense - tccttcttaatcctcttcgaacga	
HID-1γ	Sense - cccttctcttctgccctatgtgt	105
	Antisense - tccttcttaatcctcttcgaacga	
HID-1κ	Sense - actcctgtgacgggccttggaa	132
	Antisense - tccttcttaatcctcttcgaacga	
HID-S1	Sense - gaaaagaagaccagatgataat	125
	Antisense - tccttcttaatcctcttcgaacga	
HID-S2	Sense - gaaaagaagaccagatgataat	144
	Antisense - ctaggcgagagctaggaggga	
HID-E2 (Total)	Sense - gaaaagaagaccagatgataat	72
	Antisense - tccttcttaatcctcttcgaacga	
